# Correlation of a clinical activity index in comparison to frequently measured laboratory values in inflammatory bowel disease

**DOI:** 10.1007/s00384-025-04829-6

**Published:** 2025-02-19

**Authors:** Marja Rapo, Pauliina Molander, Clas-Göran af Björkesten, Suvi Pakarinen, Perttu Arkkila

**Affiliations:** 1 University of Helsinki and Helsinki University Hospital, Helsinki, Finland; 2 Abdominal Center, University of Helsinki and Helsinki University Hospital, Gastroenterology Helsinki, Finland; 3 BCB Medical Ltd, Turku, Finland

**Keywords:** Activity index, Crohn’s disease, Ulcerative colitis, Biomarkers

## Abstract

**Purpose:**

Several laboratory tests are used to monitor disease activity and possible complications in patients with inflammatory bowel disease (IBD). Due to limited resources, it is important to identify patients who benefit the most from tight laboratory testing and follow-up. We sought to assess the correlation between a symptom-based clinical activity index and commonly monitored laboratory tests in a large patient population.

**Methods:**

The Finnish IBD registry records a validated IBD symptom index questionnaire (IBD-SI) that measures disease activity and the influence of IBD on daily life in patients with ulcerative colitis (UC) and Crohn’s disease (CD). The activity index was compared with the commonly measured laboratory values of fecal calprotectin (FC), hemoglobin (Hb), ferritin, and C-reactive protein (CRP).

**Results:**

A total of 5044 IBD patients with 171,967 activity index measurement pairs were included. FC, Hb, and CRP correlated significantly with the activity index in both UC (Spearman’s *r* 0.383, −0.212, 0.175; *p* < 0.001) and CD (Spearman’s *r* 0.156, −0.176, 0.152; *p* < 0.001). No correlation between the activity index and ferritin (Spearman’s *r* 0.038 [UC], 0.005 [CD]; *p* = 0.020, *p* = 0.825) was found.

**Conclusion:**

The activity index is a useful tool in the assessment of IBD activity. Active or inactive disease can be identified better, which may be beneficial in planning more personalized follow-up strategies. Tight monitoring of disease can be better targeted to the correct patient population, and the onset of disease flare may be caught at an earlier stage.

## Introduction

Inflammatory bowel disease (IBD) is a chronic inflammatory disorder of the gastrointestinal tract. IBD includes ulcerative colitis (UC) and Crohn’s disease (CD), which are characterized by alternating relapse and remission periods. UC commonly causes mucosal inflammation in the colon, while CD may cause mural and transmural inflammation in any part of the gastrointestinal tract, leading to complications such as fistulae and abscesses [1, 2]. IBD is a significant health problem worldwide, as its prevalence has increased significantly during the twenty-first century [3]. Currently, there is no cure for IBD. The goal of treatment is to control intestinal inflammation and to prevent structural tissue damage and disease-related complications [4, 5]. Since IBD is a chronic condition, constant monitoring of symptoms and disease activity is necessary to guide clinical decisions on subsequent therapy [6].

Interest in patient-reported outcomes has increased in recent years. The Helsinki University Hospital (HUS) has developed an IBD symptom index (IBD-SI) for symptom reporting in clinical practice. The IBD-SI consists of two parts, namely an activity index score and a visual analog scale (IBD-VAS) that measures the influence of IBD on the patient’s daily life. IBD patients who have been enrolled into the IBD registry receive a text message 2 weeks before their planned appointment or remote contact with a link to the IBD-SI questionnaire. After completing the questionnaire online, the patient receives a text message indicating whether the disease is in an active state along with potential further instructions. A previous study found that the activity index correlates well with fecal calprotectin (FC) and is sensitive and specific in differentiating between remission and active inflammation in both UC and colonic CD. However, it should be noted that the study that evaluated the correlation included a relatively small number of patients (*n* = 72), and no correlation was found between the IBD-VAS and FC [7].

Although C-reactive protein (CRP) has been widely used for many years as an inflammatory biomarker to assess disease activity in patients with IBD, it has low sensitivity and specificity in detecting bowel inflammation [8, 9]. Furthermore, despite several studies suggesting that elevated CRP levels can predict the recurrence of CD, the usefulness of CRP has not yet been established due to the lack of high-accuracy data [10]. Accordingly, FC has been identified as a more reliable biomarker for monitoring inflammatory activity. Normal FC levels are an accurate predictor of sustained remission, while elevated FC levels indicate an increased risk of relapse [11–14].

The relationship between the activity index and other laboratory tests often measured from IBD patients, such as hemoglobin (Hb) and ferritin, has been studied infrequently even though anemia is a common complication associated with active IBD [15]. In addition, the relationship between the patient-reported impact of IBD on everyday life and FC has not been well established [7]. Therefore, we sought to assess the correlation between the symptom-based clinical activity index and commonly monitored laboratory tests in a large patient population to gain a better understanding of the relationship between these factors.

## Materials and methods

### Data collection

This observational cohort study included a total of 5044 patients with IBD from HUS, which is by far the largest provider of specialized medical care in Finland and covers a population of over 2.2 million people. Patient data were obtained from the Finnish IBD registry, which was developed by BCB Medical with the aim of creating a user-friendly and comprehensive patient registry that can be integrated with existing electronic health record systems for IBD patients. HUS started using this registry in 2016 to record patients with IBD and to assess disease burden with the aim of providing optimal healthcare for all IBD patients and monitoring treatment outcomes, especially in patients who are receiving advanced therapy. As the registry lacks national funding, only hospitals with allocated resources can participate in the registry. However, the administration encourages physicians to use the quality registries as part of every appointment.

All patients with an IBD-SI score recorded and laboratory tests taken within 30 days from the IBD-SI score were included in the study. Patient data from 1 January 2016 to 31 December 2023 were collected, covering a span of 8 years. Due to preserving patient information security, outlier values cannot be shown in the figures (Act of Secondary Use of Health and Social Data in Finland).

### Registry design and measures

All IBD patients at HUS are enrolled into the IBD registry. The patient’s name and address are entered into the system automatically. Healthcare personnel manually record the patient’s background information, such as sex, primary diagnosis (ICD-10), disease location, phenotype based on the Montreal classification, year of diagnosis and onset of symptoms, extraintestinal manifestations, family history of IBD (both UC and CD; in both first degree [parents/siblings] and second degree [paternal/maternal uncles/aunts and cousins] relatives), requirement of small-bowel surgery, thiopurine methyltransferase genotype, and smoking habits.

The registry subsequently automatically records medication, laboratory results, outpatient clinic visits, telephone encounters, endoscopies, radiological examinations, patient-reported outcomes (symptom questionnaire), and surgical procedures (classified according to the Nordic Classification of Surgical Procedures [NCSP]).

### Clinical assessment of disease activity by activity index

The IBD-SI questionnaire is a validated tool that contains five questions about symptoms (questions 1–5, Table 1). It generates a clinical activity index that ranges from 0 to 15 (questions 1–5, Table 1). In addition, the IBD-VAS assesses how IBD has affected the patient’s daily life during the last week (question 6, Table 1). The activity index score cutoff points determine the disease activity, with scores of 0–3 indicating remission, 4–5 indicating mildly active disease, 6–10 indicating moderately active disease, and scores 11–15 indicating severely active disease. Even during remission (according to the activity index score), the IBD-VAS can be useful in identifying those who require psychosocial support due to disease burden [7].

**Table 1:**
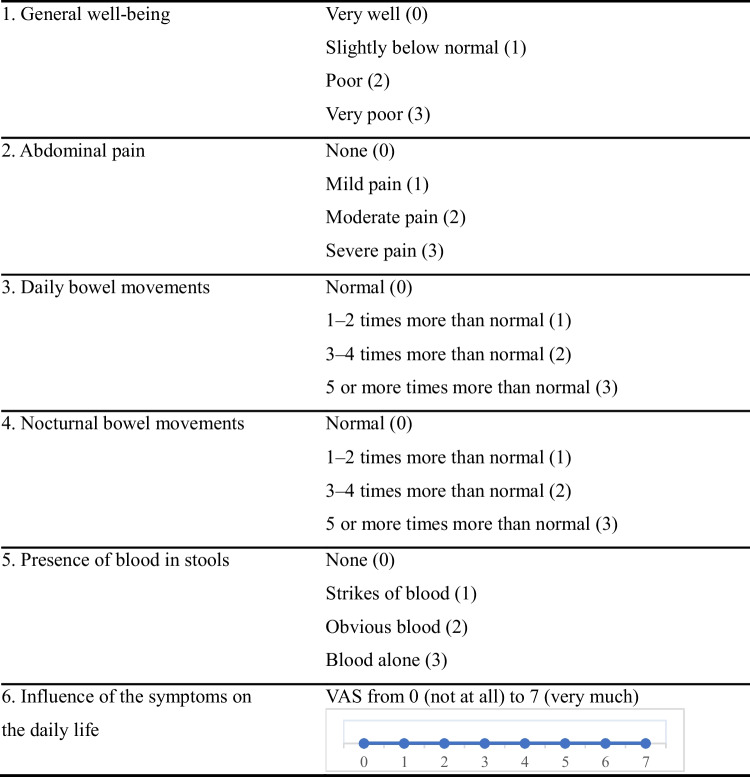
The IBD symptom index (IBD-SI) questionnaire

The questionnaire comprises two parts, a clinical activity index and a visual analog scale (IBD-VAS). The activity index ranges from 0 to 15, and the IBD-VAS ranges from 0 to 7, both of which measure the impact of IBD on the patient’s daily life. The questionnaire asks the patient to report symptoms experienced during the last 7 days

### Laboratory measurements

**Fig. 1:**
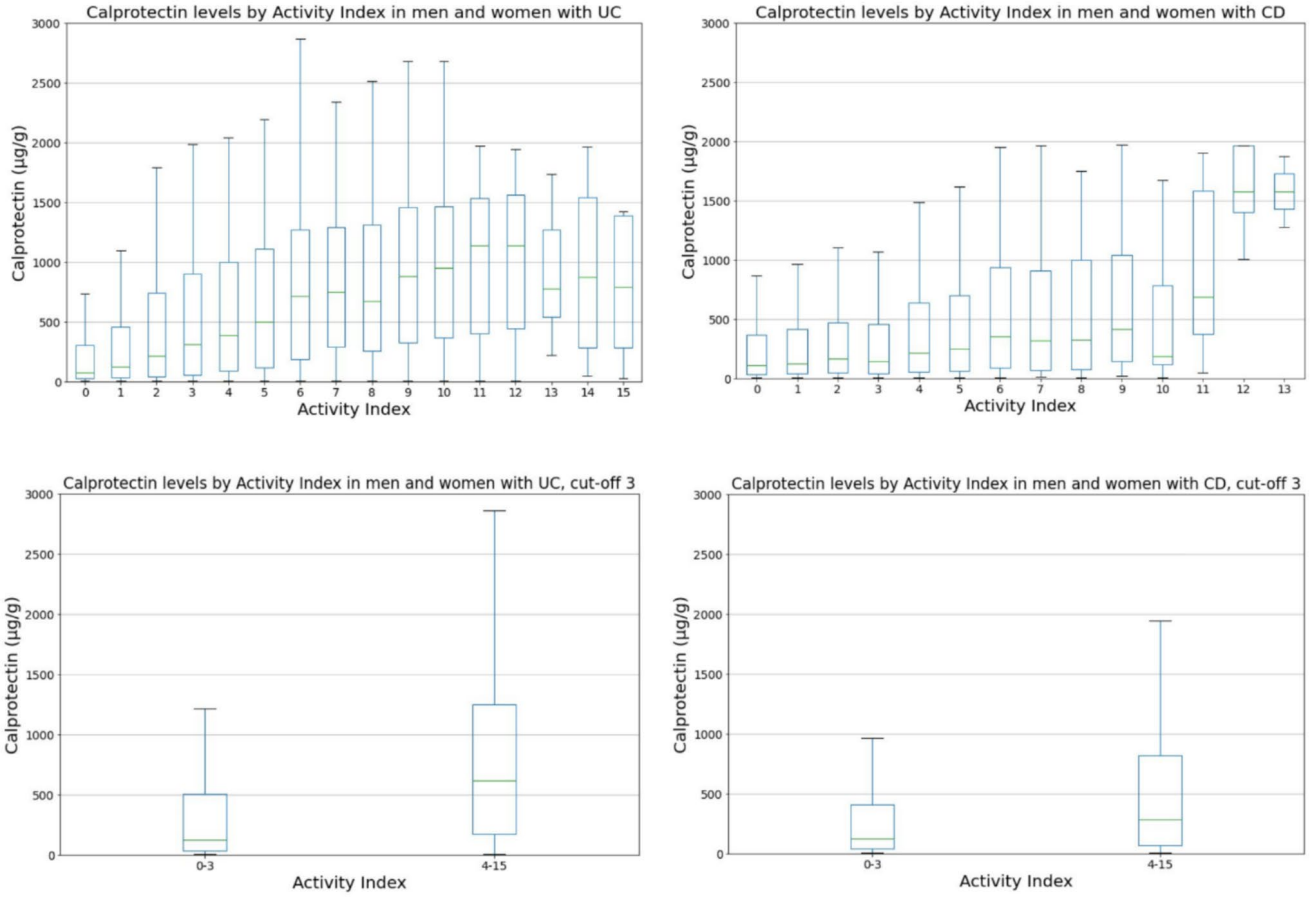
FC levels by activity index in patients with UC and CD (*p* < 0.001 and *p* < 0.001 with cut-off 3). FC, fecal calprotectin; UC, ulcerative colitis; CD, Crohn’s disease

**Fig. 2:**
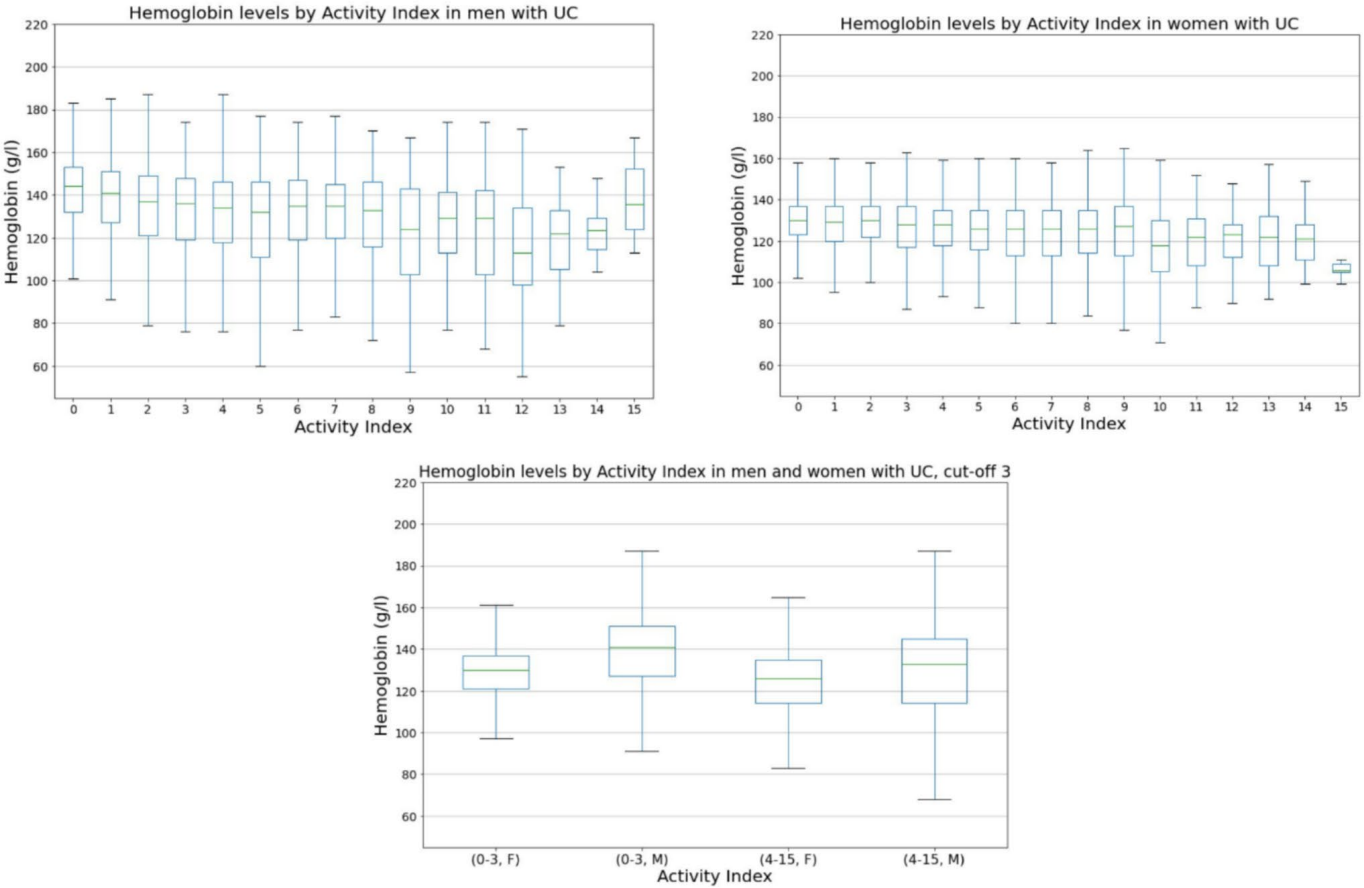
Hb levels by activity index in patients with UC (*p* < 0.001 with cut-off 3). Hb, hemoglobin; UC, ulcerative colitis; F, females; M, males

**Fig. 3:**
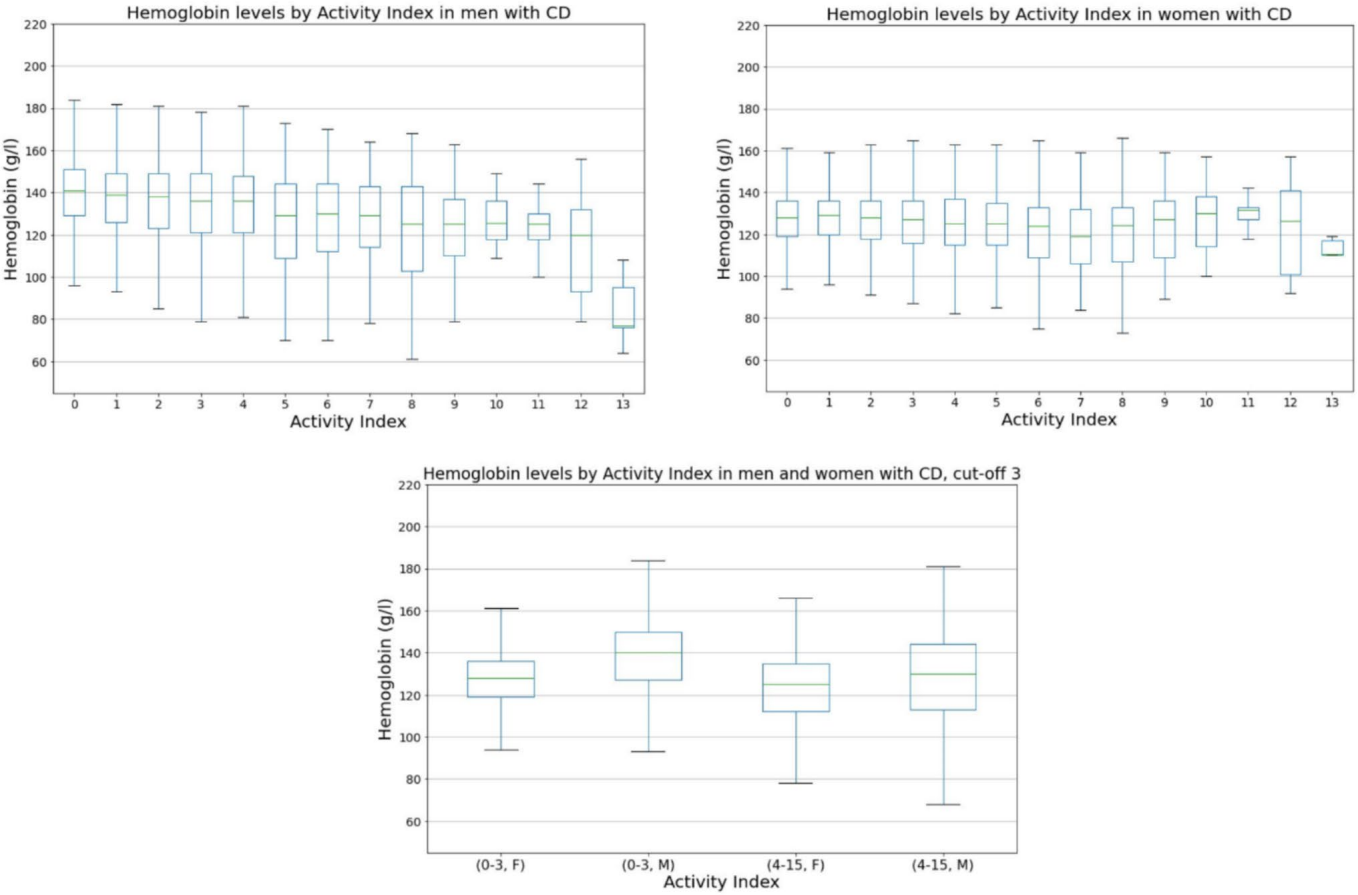
Hb levels by activity index in patients with CD (*p* < 0.001 with cut-off 3). Hb, hemoglobin; CD, Crohn’s disease; F, females; M, males

**Fig. 4:**
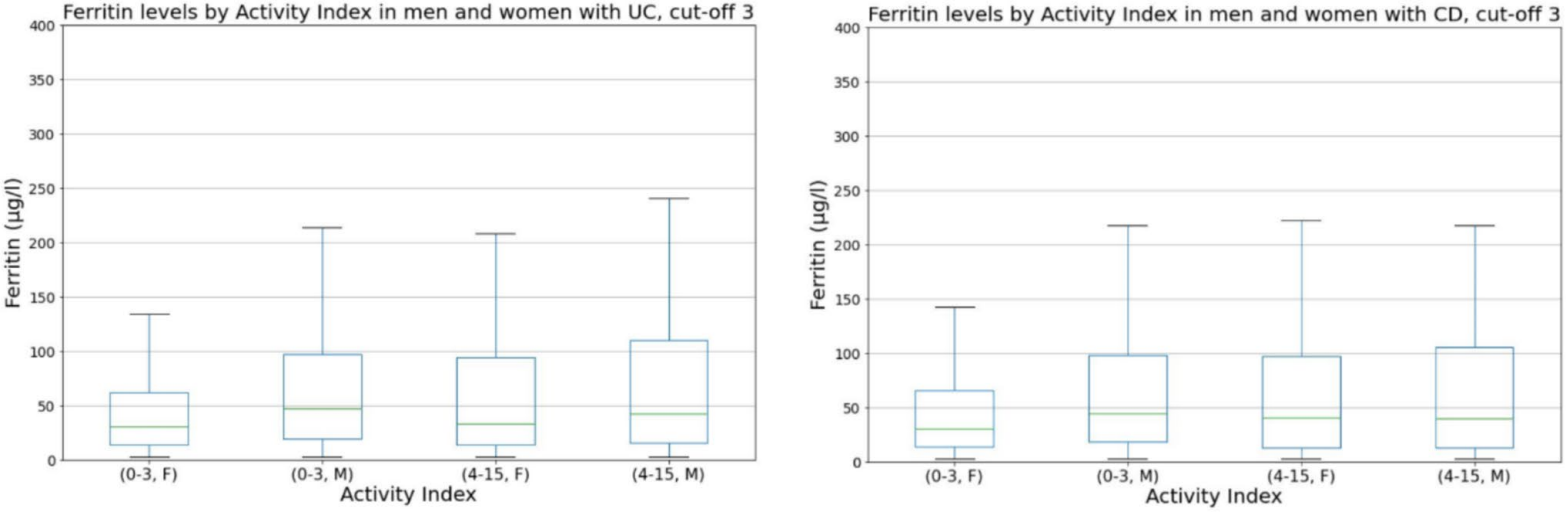
Ferritin levels by activity index in patients with UC (*p* = 0.020 with cut-off 3) and CD (*p* = 0.825 with cut-off 3). UC, ulcerative colitis; CD, Crohn’s disease; F, females; M, males

**Fig. 5:**
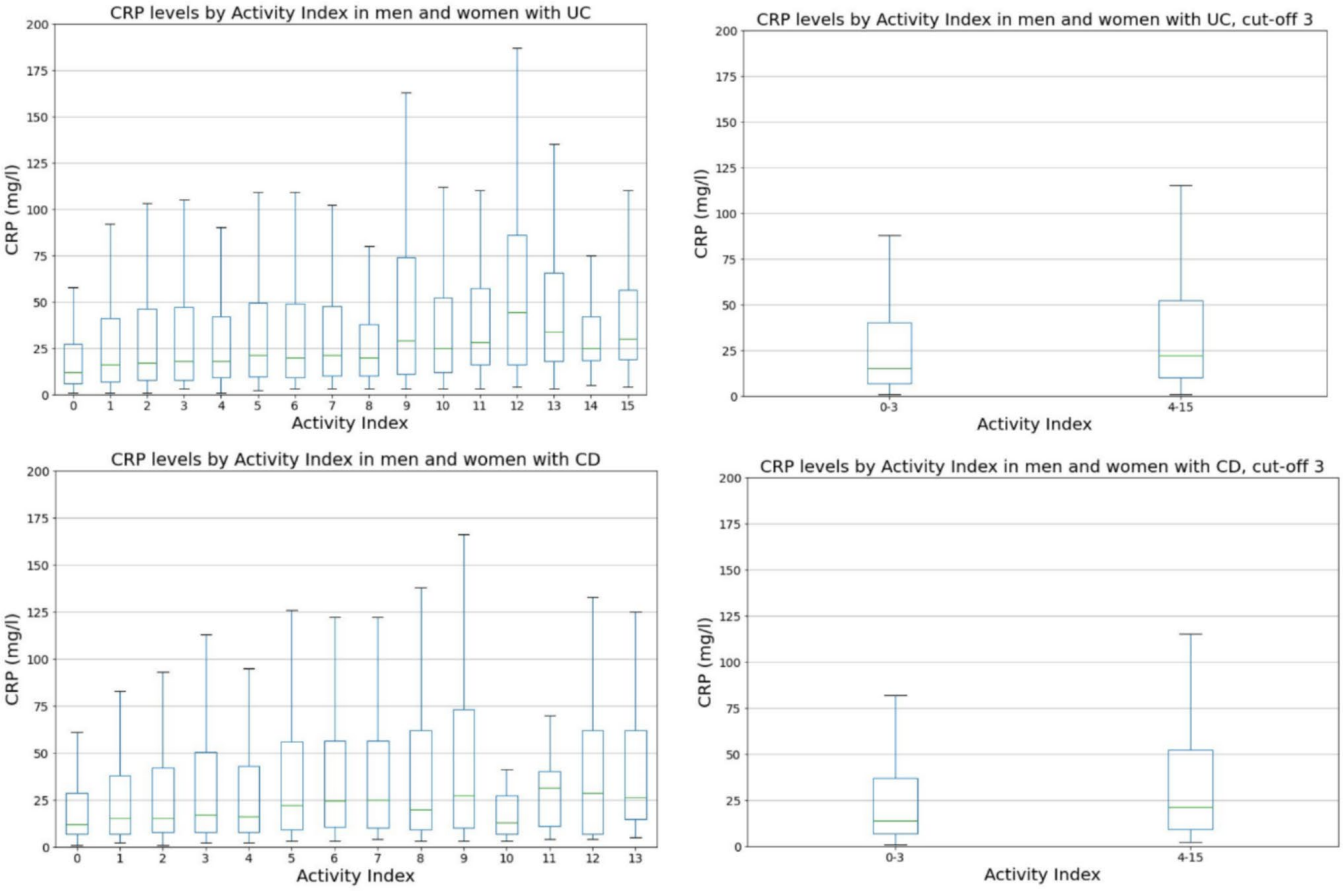
CRP levels by activity index in patients with UC and CD (*p* < 0.001 and *p* < 0.001 with cut-off 3). CRP, C-reactive protein; UC, ulcerative colitis; CD, Crohn’s disease

We compared the activity index with four different laboratory parameters (FC, Hb, ferritin, CRP). We visualized these comparisons using graphs (Figs. 1, 2, 3, 4, and 5). Normal FC values were considered <200 µg/g. Our laboratory cutoff points defined normal values for the other laboratory parameters (CRP <4 mg/l; ferritin >15 µg/l for women and >20 µg/l for men; hemoglobin <117 g/l for women and <134 g/l for men).

### Statistical analyses

Statistical analyses were conducted using Microsoft Azure Databricks and Python version 3.8. For descriptive analyses, mean, standard deviation (SD), median, range, and mean absolute deviation (MAD) were calculated for continuous variables. Median was used for asymmetrically distributed values (FC and CRP). Values <4 mg/l for CRP are not routinely collected by laboratory systems and were thus excluded from the analysis. For comparisons, two-tailed Mann-Whitney U test was used, with p-values < 0.001 considered statistically significant. Also, Spearman's rank order correlations (r) were calculated, with p-values < 0.001 considered as statistically significant.

### Ethical statement

The study was approved by the institutional review board at Helsinki University Hospital (reference number HUS/23/2024). As the persons involved in the study were not contacted and the study did not affect the patient’s treatment, informed consent was not required by Finnish legislation. The study was conducted in accordance with the latest version of the Helsinki Declaration, Good Pharmacoepidemiology Practices, and Data Protection Directive.

## Results

A total of 5044 IBD patients (3114 UC [62%], 1930 CD [38%]) with 171,967 activity index measurement pairs were included. Of these, 56% were male; the mean age of the whole cohort at the date of answer was 36 years (range 0–96 years). The demographic and disease characteristics of the patients included in the study are summarized in Table 2.

**Table 2:** Demographic and clinical data of the cohort

Total number of patients, *n* = 5044	UC, *n* = 3114	CD, *n* = 1930
Sex, male (%)	1779 (57.1)	1027 (53.2)
Disease duration (years; median, range)	8 (1–63)	9 (0–55)
Montreal classification for UC, *n* (%)		
E1	140 (8.8)	
E2	409 (25.6)	
E3	1048 (65.6)	
Montreal classification for CD, *n* (%)		
A1		356 (28.1)
A2		651 (51.4)
A3		260 (20.5)
L1		322 (26.2)
L2		244 (19.9)
L3		554 (45.1)
L4		35 (2.8)
L1+L4		20 (1.6)
L2+L4		16 (1.3)
L3+L4		38 (3.1)
B1		458 (48.8)
B2		391 (41.7)
B3		89 (9.5)

*UC* ulcerative colitis, *CD* Crohn’s disease

During the study period, 29,908 activity index values, 28,239 FC values, 36,139 Hb values, 8232 ferritin values, and 50,980 CRP values were available.

The median FC was 126 µg/l in UC patients with self-reported inactive disease (activity index 0–3); the corresponding value was 621 µg/l in UC patients with self-reported active disease (activity index 4–15) (Fig. 1). The median FC values for CD patients in self-reported/clinical remission and with active disease were 126 µg/l and 280 µg/l, respectively. Significant differences were found both in UC and CD (*p* < 0.001 and *p* < 0.001), which indicates a strong association between high FC and self-reported/clinically active disease.

The mean Hb was 133.3 g/l in UC patients with inactive disease (activity index 0–3). The corresponding mean value was 126.4 g/l in UC patients with active disease (activity index 4–15) (Fig. 2), for a difference of 6.9 g/l (*p* < 0.001). In CD patients with inactive and active disease, the mean Hb values were 132.1 and 126.1 g/l (Fig. 3), respectively, for a difference of 7.0 g/l (*p* < 0.001). The differences in Hb levels of this extent are clinically significant and indicate that high activity is associated with decreased Hb. Notably, active disease seems to lead to Hb levels below the reference range in many cases. There was also a difference between the sexes; 41% of men with IBD on average have Hb below the reference range, while the corresponding percentage of women was 25%.

When ferritin levels in inactive and active disease were compared (Fig. 4) (Spearman’s *r* 0.038 [UC], 0.005 [CD]; *p* = 0.020, *p* = 0.825), both UC and CD patients with active disease had only marginally higher mean ferritin levels than patients with inactive disease (70.7 µg/l vs. 89.5 µg/l for UC, 66.7 µg/l vs. 75.9 µg/l for CD). In addition, individual patient ferritin levels are independent of disease activity; thus, no correlation between ferritin and activity is evident.

Median CRP was 15.0 mg/l in UC patients with inactive disease; the corresponding value in UC patients with active disease was 23.0 mg/l (*p* < 0.001). The corresponding values in CD patients were 14.0 mg/l and 21.0 mg/l (*p* < 0.001) (Fig. 5).

## Discussion

The inflammatory activity of IBD is regularly monitored through laboratory tests, endoscopic and radiological measures, and non-invasive surrogate markers. Among these markers, FC is the most used [16]. The findings of our study demonstrate that the self-reported simple activity index and FC correlate well both in UC and CD. As expected, a weak correlation between Hb and the activity index and CRP and the activity index was found. However, based on our study, the commonly measured ferritin did not correlate with active disease.

Various other activity indices have been previously developed, such as the simplified endoscopic activity score (SES-CD) for CD [17] and the UC endoscopic index of severity (UCEIS) for UC [18]. The Mayo score, developed in 1987, is a widely used combined endoscopic and clinical scale used to assess UC severity [19]. Other commonly used clinical indices are the Simple Clinical Colitis Activity Index (SCCAI) for UC and the CD Activity Index (CDAI) and the Harvey Bradshaw Index for CD [20]. However, these activity scores are either only symptom-based, too complex for clinical use, or cannot be completed by patients themselves, hence the need for a self-reported digital symptom questionnaire [21, 22].

One of the main long-term goals of IBD treatment established by an expert consensus (selecting therapeutic targets in inflammatory bowel disease, STRIDE) is to restore quality of life and eliminate disability [23]. To achieve this goal, it is recommended to frequently assess patient-reported outcome measures (PROMs) and patient-reported experience measures (PREMs) in addition to laboratory, imaging, and endoscopy observations. Previous studies have shown that many PROMs are moderately accurate in detecting mucosal inflammation and FC in UC patients, but less accurate or not correlated in patients with CD. The validation study for the PROM used in our registry found that the activity index significantly correlates between histological activity and FC, with the correlation being stronger in UC patients than in CD patients [7]. This study revealed that if the patient-reported activity is low, it is likely that FC is also low, and the patient does not have severe anemia. Thus, clinical follow-up intervals could be increased, and routine laboratory tests could possibly be reduced.

It is often recommended to monitor CRP in follow-up of IBD patients. However, CRP usually only increases when the inflammation is severe and clinically detectable. Furthermore, normal CRP levels can be observed in patients even with active IBD [8, 9]. Based on earlier studies, elevated CRP levels in CD are associated with clinical disease activity and endoscopic inflammation [24], and early normalization of CRP predicts therapeutic response in CD. The effect of tight control management on CD (CALM) study [25] was the first randomized trial to demonstrate that in patients with early CD, therapy based on biochemical targets in addition to clinical targets (tight control arm) is associated with higher endoscopic remission at 1 year compared with therapy based on clinical targets alone (clinical management arm). In our study, we found a marginal increase in CRP in active disease, with levels far above the reference in both clinically active and inactive disease. However, CRP is not a specific marker of intestinal inflammation, and therefore, other drivers for elevated CRP should be considered. Moreover, as most patients with UC do not have elevated CRP levels, the applicability of CRP measurements in UC as an isolated monitoring tool is still questionable.

Anemia is associated with reduced quality of life and is a common cause of frequent hospitalization, delay of discharge, and increased healthcare burden in IBD patients [26]. Serum ferritin is the most accurate predictor of bodily iron stores. However, as ferritin is an acute-phase reactant, it can be falsely normal or elevated in inflammatory conditions [27]. Therefore, in the presence of inflammation, a serum ferritin level of up to 100 µg/l may still be consistent with iron deficiency. Importantly, correction of anemia in IBD patients improves health-related quality of life and overall well-being, and this improvement is independent of clinical disease activity [28]. Ferritin is often monitored on a regular basis even though it does not correlate well with IBD-related inflammation. A French study [29] showed that iron deficiency was detected in about a quarter of patients, and its prevalence was higher in women and in subjects with severe IBD activity or concomitant anemia. While inflammation increases ferritin levels, intestinal bleeding causes ferritin levels to decrease [30]. Our study revealed that individual patient ferritin levels are independent of activity. However, low Hb seems to be associated with active disease. Thus, it may be preferable to target ferritin monitoring to clearly anemic patients.

The major strengths of this study were the wide registry-based study cohort with long-term follow-up of patients and the availability of several different laboratory values. Due to the size of the cohort (5044 IBD patients), the results are both more likely applicable to real-world clinical settings and generalizable to a broader population. A large study cohort also increased the probability for detection of smaller effect sizes with greater statistical significance.

Some limitations should be acknowledged. A major limitation is associated with retrospective data collection. Differences in the quality of data recording (e.g., better documentation in certain subgroups) may lead to misleading information. However, it can be assumed that a large data set will reduce the potential error caused by these factors. Another weakness of registry-based studies is that it is difficult to consider all possible confounding factors, especially if not all relevant variables are recorded in the register. Moreover, even though the size of this cohort was large, the data were primarily from specialized care. Further studies with a more diverse study cohort may be beneficial to obtain results applicable to all IBD patients.

In conclusion, this study suggests that the activity index facilitates follow-up of IBD activity. IBD patients may undergo laboratory tests several times a year, regardless of clinical activity. Based on this study, closer monitoring of the disease can be better targeted to the right patients and the onset of disease flare may be caught at an earlier stage. At the same time, unnecessary laboratory tests can be reduced.

## Data Availability

The data that support the findings of this study are not publicly available due to reasons of sensitivity. Data are located at the Finnish IBD registry and is available from the authors upon reasonable request and with permission of the Finnish IBD registry.
